# An Efficient 5G Data Plan Approach Based on Partially Distributed Mobility Architecture

**DOI:** 10.3390/s22010349

**Published:** 2022-01-04

**Authors:** Mohammad Al Shinwan, Laith Abualigah, Trong-Dinh Huy, Ahmed Younes Shdefat, Maryam Altalhi, Chulsoo Kim, Shaker El-Sappagh, Mohamed Abd Elaziz, Kyung Sup Kwak

**Affiliations:** 1 Faculty of Computer Science and Informatics, Amman Arab University, Amman 11953, Jordan; Aligah.2020@gmail.com; 2 School of Computer Sciences, Universiti Sains Malaysia, Gelugor 11800, Malaysia; 3 Department of Computer Engineering, Inje University, Gimhae 633165, Korea; tronghuy.fetelpro@gmail.com (T.-D.H.); charles@inje.ac.kr (C.K.); 4 IST/TNT Department, College of Engineering and Technology, American University of the Middle East, Egaila 15453, Kuwait; ahmed.shdefat@aum.edu.kw; 5 Department of Management Information System, College of Business Administration, Taif University, Taif 21944, Saudi Arabia; marem.m@tu.edu.sa; 6 Faculty of Computer Science and Engineering, Galala University, Suez 435611, Egypt; sh.elsappagh@gmail.com (S.E.-S.); meahmed@zu.edu.eg (M.A.E.); 7 Information Systems Department, Faculty of Computers and Artificial Intelligence, Benha University, Banha 13518, Egypt; 8 Department of Mathematics, Faculty of Science, Zagazig University, Zagazig 44519, Egypt; 9 Artificial Intelligence Research Center (AIRC), Ajman University, Ajman 346, United Arab Emirates; 10 School of Computer Science and Robotics, Tomsk Polytechnic University, 634050 Tomsk, Russia; 11 Department of Information and Communication Engineering, Inha University, Incheon 22212, Korea

**Keywords:** 6G network, 5G network, 4G network, mobile core network, partially distributed mobility

## Abstract

Reaching a flat network is the main target of future evolved packet core for the 5G mobile networks. The current 4th generation core network is centralized architecture, including Serving Gateway and Packet-data-network Gateway; both act as mobility and IP anchors. However, this architecture suffers from non-optimal routing and intolerable latency due to many control messages. To overcome these challenges, we propose a partially distributed architecture for 5th generation networks, such that the control plane and data plane are fully decoupled. The proposed architecture is based on including a node Multi-session Gateway to merge the mobility and IP anchor gateway functionality. This work presented a control entity with the full implementation of the control plane to achieve an optimal flat network architecture. The impact of the proposed evolved packet Core structure in attachment, data delivery, and mobility procedures is validated through simulation. Several experiments were carried out by using NS-3 simulation to validate the results of the proposed architecture. The Numerical analysis is evaluated in terms of total transmission delay, inter and intra handover delay, queuing delay, and total attachment time. Simulation results show that the proposed architecture performance-enhanced end-to-end latency over the legacy architecture.

## 1. Introduction

In recent years, the increase in mobile traffic exerted pressure on mobile operators to re-engineer next-generation core networks by proposing flat-network architecture to provide a scalable solution for billions of devices [1,2,3]. Future data traffic is expected to double every year in the next five years. More than 100 billion connections of Internet of Things (IoT) devices are envisioned to be deployed by various operators [4,5,6]. This creates numerous new challenges for future mobile communication technology operators to handle such data traffic and enhance network capacity. Next-generation (5G) mobile networks aim to provide faster speed and a higher number of connected users to overcome such challenges [7,8].

Mobile operators and industry partners are collaborating to address such challenges in the near future [9,10]. The main two challenges to address are: firstly, enhancing the network capacity for the vast number of connected devices and, secondly, efficiently handling all data traffic passing through the network. Many approaches have been proposed for the first challenge, including device-to-device communication and radio resource management. Small cell technology [11], which utilizes available resources to provide better coverage and data rates, which is considered as a promising approach [12]. Several methods have been suggested for the second challenge to overcome the centralized core network and hierarchical architecture adjustment. Therefore, it is essential to enhance the current architecture by proposing a flat core network architecture to handle the many devices connections, and big data generators such as IoT [13,14].

**Figure 1:**
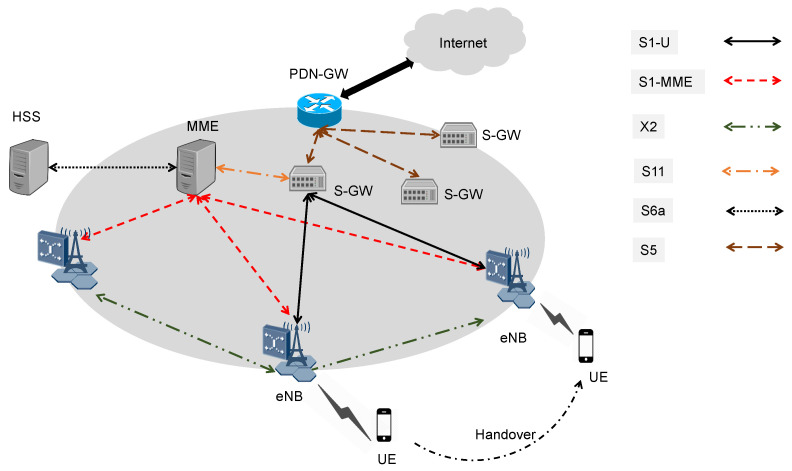
The Evolved Packet Core (EPC) model for 4th generation (4G) mobile networks.

The current 4th generation (4G) is based on the Evolved Packet System (EPS), which has a flat IP-based architecture as shown in Figure 1 [15,16]. Such architecture is composed of two main components the Evolved Universal Terrestrial Radio Access Network (E-UTRAN) and the Evolved Packet Core (EPC) [17]. E-UTRAN is consists of base stations eNodeB (eNB). On the other hand, The EPC consists of Management Mobility Entity (MME), anchor mobility Serving Gateway (S-GW), Packet-data-network gateway (P-GW), and Home Subscription Server (HSS). For routing data from a P-GW to the User Equipment (UE), a combination of S1-U, S5/S8, and X2 bearer are used. S1-U is a connection between eNB and S-GW, while S5/S8 delivers traffic from S-GW to P-GW, and X2 is a temporary connection in case of mobility. In the 4G data plane architecture, S-GW acts as a mobility anchor and forwards packets between eNB and P-GW. Furthermore, P-GW acts as an edge router, enabling UE to connect to the Internet. Additionally, the primary function of P-GW is traffic directing, packet filtering, and IP allocation. MME manages and controls all procedures in a centralized manner. Finally, the data delivery procedures, including initial attach and mobility management, are based on the concept of GPRS tunneling protocol (GTP) [18,19].

Such centralized architecture has several challenges. Firstly, in non-optimal routing, data packets in uplink and downlink have to route via mobility anchor; this might not be the shortest path between P-GW and UE. Moreover, packets still need to go through the core network via the mobility anchor with longer distances, increasing the end-to-end delay. Secondly, managing both data and control traffic in S-GW and P-GW increase the packet overhead, signaling, and producing a single point of failure.

Several researchers attempt to enhance the EPC architecture by proposing Software Defined Networking (SDN) technology. For instance, Refs. [20,21], the authors decomposed and classified the functions according to their impact on the control plane and data plane processing while proposing an SDN network element using the OpenFlow protocol. In [22], the authors suggested OpenFlow-based architecture by merging control protocols that run on S1-MME and S11, several other proposals can be found in [23,24]. All such proposals show SDN-based architecture splitting control and data plane. They analyzed the procedures necessary for the entire OpenFlow-enabled LTE/EPC.Although the results with signaling load are good, the proposed architectures are still based on centralized mobility. Therefore, we propose a partially distributed EPC architecture to enhance end-to-end latency.Although the results with signaling load are good, their proposal architectures are still based on centralized mobility. Therefore, we are proposing a partially distributed EPC architecture to enhance end-to-end latency.

Distributed Mobility Management (DMM) is an exciting approach attracting much research from both academia and industry. The results in [25,26] indicated that DMM is a promising solution to the challenge of handling a vast number of data traffic over the mobile network. This paper [27] shows that there are two types of design for DMM, partially distributed and fully distributed. In the partially distributed scheme, the control plane is centralized while the data plane is distributed. However, the entire distribution scheme distributes both the control plane and the data plane. The author concluded that a fully distributed approach is valuable for limited deployments, such as local to metropolitan areas, but not suitable for global area networks. Thus, partial distribution becomes a primary key in our proposed 5G architecture.

In [28] the authors proposed two solutions for a partially distributed approach; the first solution includes PMIPv6-based DMM based modifying classical Proxy Mobile IPv6 protocol. Second, it follows a Software-Defined Networking called SDN-based DMM. While [29] presents a proxy mobile IPv6 based on DMM and presents an evaluation of SDN network and DMM solutions.

Most of the above methods show that the PMIPv6-based DMM concept and the user’s IP address are assigned and unchanged during mobility situations. The IP layer is unaware of the U.E. mobility.

In this paper [30], a mobility management model is proposed to improve the handover performance between the 4g and 5g networks. The proposed work employs dual connectivity for mobility management since dual connectivity has a minor handover delay compared to standard hard handover.. Based on the application-specific approach, the authors proposed a data split method between 4G and 5G RATs. MR Palas et al. propose mobility management method to optimize the handover process based on an improved Multi-Objective optimization (MMO) approach using Ratio Analysis (R.A.) and the Q-learning method, namely E-MOORA. The proposed work combines the enhanced entropy weighting method with the MMO by R.A. The results show that the E-MOORA approach decreases ranking anomaly during the handover process to select the target cell. Furthermore, to satisfy the user QoS demands, the Q-learning method is employed to choose the best triggering points to minimize the impact of frequent undesirable handovers [31]. Other optimization techniques that can be used are founded in [32,33,34].

The authors in [35] propose an approach that includes two algorithms to select the suitable radio access network. One of these algorithms is placed at the user equipment and the second algorithm in the core network. This approach aims to satisfy the users regarding security, quality of service, cost, and energy consumption. This paper [36] introduces a new core architecture based on eliminating the SGW and different interfaces such as S5/S8. Then suggest communicating the S1- and S11 to the PGW directly. In this paper [37], a new 4G architecture is proposed based on combining the PGW and SGW to functions as one network node. The proposed node is distributed in the backbone mobile network to manage mobility. Further, the MME node assigns the user equipment’s IP address and acts as a mobility anchor. In [38], the IP-in-IP protocol is proposed for data transmission and mobility management in the mobile core network instead of implementing the GTP protocol.

The 3gpp technical reports and standards described four main solutions for separating the user plane (UP) and control plane (C.P.) for the EPC nodes. These solutions have their drawbacks on other entities in the core network, such as MME. Therefore, in our proposal, we are distributing the functionality of UP while keeping the C.P. intact. The UP functionality is distributed during the PDN establishment, changing the UP session identity and relocating UP functionality based on the U.E. mobility. Our proposed architecture shall eliminate the additional signaling requirements as described in [39,40,41]

In terms of handover, different approaches were proposed for Ultra-dense networks (UDNs) based on the trigger time and eNB cooperation set to reduce the handover procedure time. Sun, Wen, et al. propose to use the making coordinated multipoint (CoMP) protocol to transmission approach. The solution aims to track the user movement intelligently to assigned small or microcell by gauging the dwell time. Further, the authors suggest an improved movement-aware CoMP handover called iMACH to improve the reliability. The numerical and simulation results show an out-performance regarding the handover probability, coverage, and throughput [42]. This paper [43] presents a multi-attribute mobility handover approach based on the throughput necessity and link reliability to enhance the quality of experience (QoE) and quality of service (QoS). Furthermore, to guarantee the QoS requirement, the lowest throughput is considered. The UE connects to the target cell with the highest priority, which is computed according to related weights, QoS index, and link index. The experimental results show the superiority in terms of handover delay and throughput.

Therefore, we proposed an SDN-based DMM architecture for the 5G mobile core network in this research. The proposed architecture comprises a centralizing control function with SDN technology support and distributing the user data plane. This proposal aims to simplify signaling messages and eliminate the number of entities in the data path, thus, reducing the total latency. We focus on analyzing necessary data plane procedures, including initial attachment, data delivery, handover in intra-gateway and inter-gateway situations. Experiments are conducted on several scenarios using NS-3 simulation to validate the performance of the proposed architecture. Firstly, we build new network entities representing the mobile core, such as Mobile Control Entity, which combines the MME, S-GW, and P-GW, and consists of several control entities to avoid a single point of failure. A new Mobility Gateway acts also as anchor mobility inside the network and routing IP packets to the Internet. Secondly, implement control and data plane into the corresponding interface. The proposed SDN-based DMM architecture obtained better results in terms of total transmission delay, inter and intra handover delay, queuing delay, and total attachment time. The Numerical analysis and simulation results showed that the proposed SDN-based DMM architecture performance-enhanced end-to-end latency over the legacy architecture.

The rest of this paper is presented as follows, and Section 2 provides our proposed 5G architecture and descriptions. In Section 3, the details of how the new procedures work are given. Section 4 describes the simulation environment and results. Finally, we conclude this material in Section 5.

## 2. Proposed 5G Network Architecture

**Figure 2:**
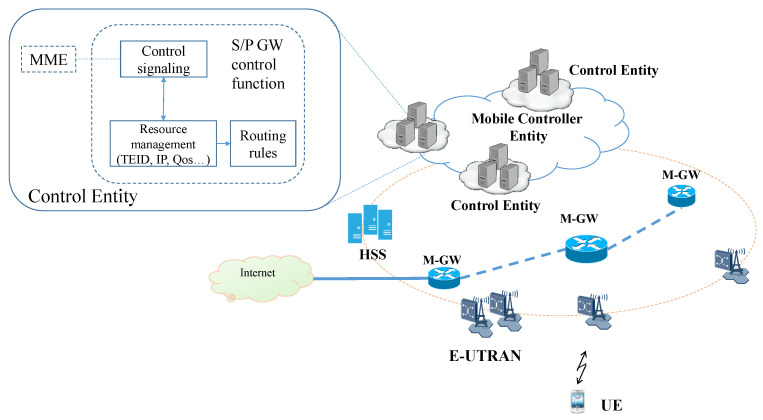
Proposed partially distributed packet core for 5G network.

The proposed 5G Architecture is illustrated in Figure 2; to satisfy the demand of the partially distributed concept, the control protocols running on S-GW (SGW-C) and P-GW (PGW-C) to MME will be removed [44]. The SGW-C, PGW-C, and MME combined in one entity, called Mobile Control Entity (MCE). MCE focuses on both management and control EPC core. The functionalities of the centralized control plane and distributed data plane are detailed below.

MCE is the central control unit that triggers a control signaling message and responds to the corresponding functions accordingly. It is responsible for U.E. authentication and authorization, tracking U.E. location, Gateway selection, and bearer control. Additionally, MCE is establishing a data-plane session by assigning the tunnel parameters such as Tunnel Endpoint Identifier (TEID), Quality Class Identifier (QCI), or Guaranteed Bit Rate (GBR) for a dedicated bearer. Besides, U.E. IP allocation function and data forwarding rules are also implemented.

The Software-Defined Networking technology [45] is realized to reach the separating control and data plane. In order to avoid a single point of failure, MCE should be divided into several Control Entity (C.E.); each C.E. manages an area covered by a group of E-UTRAN and Gateway. Thus, the U.E. IP address is unchanged during mobility in the same C.E. domain. However, when U.E. moves to a new C.E. domain, the new C.E. will assign a new IP address, the management of IP addresses for the DMM concept is presented in [21].

In a centralized LTE architecture, the Mobility Management Entity (MME) is responsible for tracking area updates TAU and paging procedures. With the massive increase in U.E. equipment and the envisioned IoT deployment, the signaling generated from TAU and paging procedure leads to a high volume of signaling traffic which hinders the performance of MME. Therefore, we propose a distributed data plane based on a non-anchor concept that relies on a more significant number of the gateway. Thus, the packet can be routed flexibly to the closest node, which results in reduced latency. Our proposal includes only One M-GW in charge of anchor mobility inside the core network and routing IP packets to the Internet. C.E. will allocate the M-GW depending on the distance from M-GW to U.E.’s current attached location. M-GW’s role is to receive data plan routing rules from C.E. and then connect to the corresponding nodes. Furthermore, the gateway should be handled the data plane filtering and classification responsible for distinguishing traffic based on user profile and policy for uplink and downlink. The GTP tunneling protocol is kept the same as described in LTE Core networks. In this work, we keep the C.P centralized to mimic the SDN architecture without changing the whole network structure or adding new hardware devices to satisfy the SDN architecture. Further, the number of the C.P will be increased and distributed to keep function as mobility anchor to support the data routing and distributed data plane.

The proposed architecture comprises a separated control plane and user data; this architecture will provide the operators with an efficient and intelligent network to solve the 5G problems. Decoupling the control plane and user plane will affect the hardware infrastructure by combining the S-GW and P-GW, which will significantly reduce the cost of deployment and development.

## 3. The Proposed 5G Procedure

### 3.1. Initial Attachment

**Figure 3:**
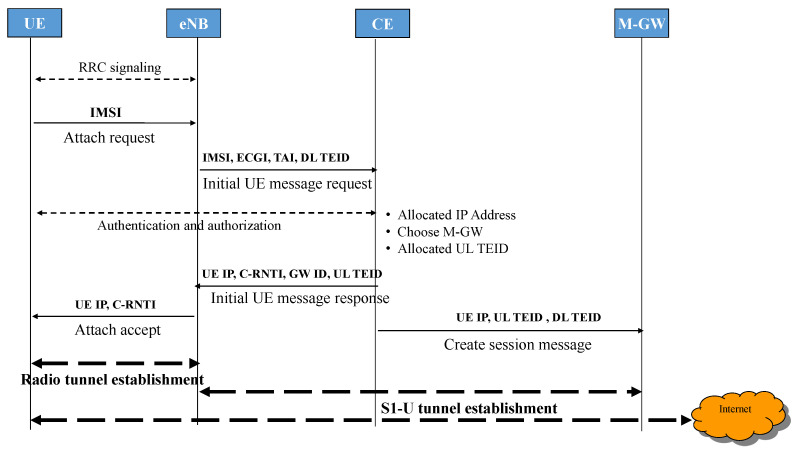
The initial attach procedure in the proposed 5G network.

When U.E. is turned on, in legacy LTE architecture, the initial attachment occurs to register the user to the network and establish a PDN connection as described in [46]. Then the UE establishes a connection with the eNB as a synchronization procedure and data delivery. The eNB passes these connection messages to the MME to update the location of the UE to HSS. Then the transmission path will be created after the MME inform the SGW to generate that path. Now, the S-GW plays the mobility anchor’s role by modifying the bearer with P-GW and letting the MME respond to the UE and accept its attached request. Finally, the UE sends an attached message to the MME to pass to S-GW to modify the bearer. However, the attachment procedure in our proposed architecture is simplified due to some adjustments in the network architecture. First, session activation in the gateway is eliminated, the U.E. registers its information and gets the IP address from the C.E. in the network. Now U.E. only needs to reach C.E. to obtain all necessary information, and therefore, the total time for attachment is reduced. Second, one M-GW can reduce the number of steps to establish a tunnel from U.E. to G.W. if U.E. has packets to be transmitted or received. The attached procedure is detailed in Figure 3 and described as follows:

The U.E. sends an Attach Request message including International Mobile Subscriber Identity (IMSI) to eNB. Then eNB forwards Attach Request message to C.E. through the Initial U.E. context message after adding other parameters like Tracking Area Identifier (TAI) or E-UTRAN Cell Global Identifier (ECGI). Authentication and authorization, and location update procedures are assumed to be unchanged. As soon as these steps are successful, C.E. now allocates U.E. IP address from its IP pool, then C.E. selects the gateway closer to U.E. An Attach accept message containing M-GW information for establishing an uplink GTP tunnel (G.W. IP, UL TEID, QoS), and U.E. IP address is sent to eNB. eNB configures radio bearer to U.E. and status of U.E. change from De-registered to Registered. At the same time, C.E. also sends a Session control message with eNB information (IP eNB, DL TEID, QoS) to create a downlink GTP connection. After this step, if U.E. needs an Internet connection immediately, a trigger message will be sent, and uplink/downlink GTP tunnels between the base station and gateway will be established. This procedure is defined in the data delivery concept below.

### 3.2. Data Delivery Procedure

The U.E. can send and receive data packets through a GTP tunnel between eNB and M-GW according to the destination address The data delivery procedure is classified as Mobile host to Internet and Mobile host to Mobile host.

#### 3.2.1. Mobile Host to Internet

**Figure 4:**
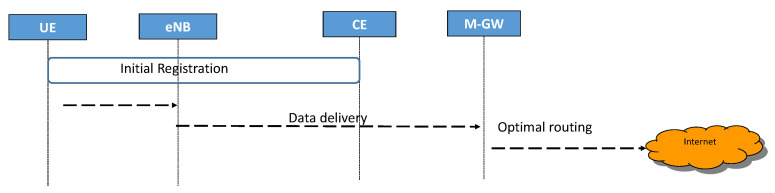
The data delivery procedure from Mobile host to the Internet in the proposed 5G concept.

In the case of data delivery between the mobile host and the Internet, as shown in Figure 4, the U.E. sends packets to eNB, eNB encapsulates the packets and forwards them to M-GW through a GTP tunnel. When M-GW receives packets, it checks the destination IP address against the routing table and makes an optimal routing decision to the Internet. This simplified procedure eliminates exchanging control messages between S-GW and P-GW. Furthermore, Our proposal simplifies S5/S8 tunnel establishment. Hence, it improves signaling and total time transmission.

#### 3.2.2. Mobile Host to Mobile Host

**Figure 5:**
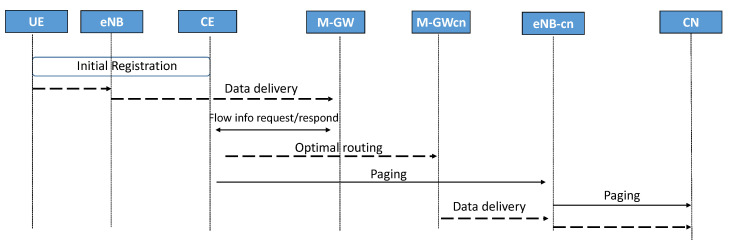
The data delivery procedure from Mobile host to Mobile host in the proposed 5G concept.

As described in Figure 5, the data delivery procedure between the mobile host and the attached core network is the communication between two mobile hosts. First, UE sends data packets through a GTP tunnel to M-GW. A decryption process occurs in M-GW to identify the destination IP address. When the M-GW decides that the destination belongs to a different network, it sends the info request message to C.E. to acquire corresponding M-GWcn IP address. Based on the C.E., the M-GW chooses a routing path to the target gateway based on its routing table. Once the M-GWcn receives the data packet, a paging procedure occurs to identify the location of the corresponding U.E. After the paging procedure, a GTP tunnel is established to deliver data packets to the C.N.

### 3.3. Intra-Gateway Handover

**Figure 6:**
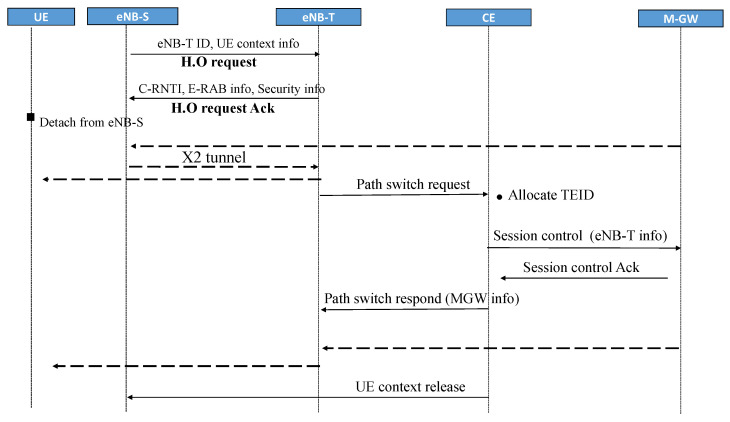
The handover without gateway relocation procedure in the proposed 5G concept.

Handover (H.O.) is a necessary procedure that maintains U.E. connectivity during the mobility inside the area of E-UTRAN. Depending on which eNB the U.E attached, the handover procedures are categorized as Intra Gateway Handover (Intra-GW H.O.) and Inter Gateway Handover (Inter-GW H.O). Intra-H.O occurs once the user moves out of the coverage area of the eNB source (eNB-S) and attaches to the target (eNB-T), which is in the same S-GW’s domain. The Intra-GW H.O. with X2 support scenario is similar to the LTE procedure [47] because E-UTRAN functions and procedures are unchanged in the proposed work. However, if both the source eNB and the target eNB belong to different S-GW’s domains, the Inter-H.O. procedure shall occur, adjustments to the signaling method may change. In this section with Figure 6, we propose a handover without a gateway relocation procedure to reduce signaling messages.

In proposed 5G network, all control functions are centralized in C.E., thus, eliminating MME involvement which simplified the H.O procedures even further. When the C.E. receives a path switch request from the target eNB, it allocates the necessary information of gateway (including TEID, QoS), adds parameters of the target eNB, and forwards them to M-GW through a session control message. After that, a Path switch response message, enclosed gateway’s information, is sent to the target eNB. A GTP tunnel is created to deliver packets from the target eNB to M-GW.

### 3.4. Inter-Gateway Handover

**Figure 7:**
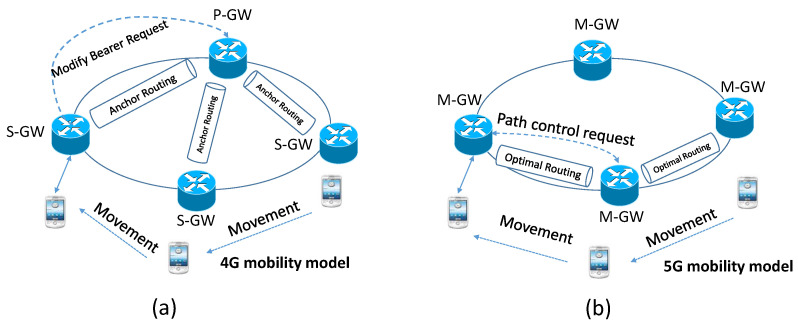
The mobility with gateway relocation in (**a**) 4G and (**b**) proposed 5G concept.

**Figure 8:**
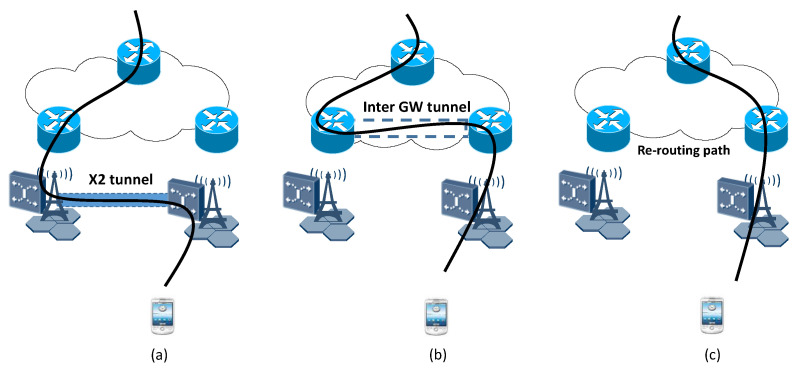
Three main steps of handover procedure with gateway relocation in proposed 5G concept: (**a**) establishing X2 tunnel, (**b**) inter M-GW tunnel, and (**c**) switching destination GW.

Inter-gateway handover occurs between a source eNB and a target eNB when the U.E. moves to a new gateway’s domain. Deploying distributed architecture is a critical step in the suggested network to provide flat network architecture. Figure 7a illustrates the mobility situation in 4G architecture, While our proposed architecture is illustrated in Figure 7b. The signaling communications during handover have been reduced immensely by eliminating the involvement of P-GW. In our proposed 5G concept with distributed M-GWs, a direct communications connection between the M-GWs, as illustrated in Figure 8, can be established. There are three main steps during the handover procedure, including establishing X2 tunnel, inter M-GW tunnel, and switching destination G.W., shown in Figure 8.

The X2 tunnel establishment is similar to traditional LTE/EPC. The source eNB chooses the target eNB and decides to handover. This decision is based on the neighbor cell list and information on the signal strength after exchanging measurement report messages to other eNBs, by preparing X2 signaling and sending a Handover request message to the target eNB. The target eNB replies with an acknowledgment that it can serve U.E. An X2 tunnel is established to the target eNB to buffer data. The U.E. starts the detach procedure from the source eNB and attaches it to the target eNB. After the attachment, buffered data will be forwarded to U.E. through a radio connection from the source eNB.

The most significant modification of the proposed scenario is inter-GW tunneling. While in the 4g network, the S-GW has to establish a GTP tunnel to P-GW by sending a modified bearer request message. Instead, in the proposed network, the packets are delivered directly through the proposed 5G concept inter-tunnel from the source M-GW (MGW-S) to the target M-GW (MGW-T). While U.E. moves to a new eNB belonging to the new M-GW, C.E. receives a path switch request from the target eNB, depending on the target M-GW. The C.E. allocates parameters and distributes the forward data plane to the corresponding gateway and eNB. GTP protocol has remained for tunneling between E-UTRAN and Core network devices. After establishing the inter-GW tunnel, packet routing in the optimal path through the direct tunnel, thus reducing end-to-end latency and enhancing performance in the core layer.

The final step is re-routing to the target M-GW. This scenario assumes packets are routed initially to the source M-GW from M-GWn located close to the source M-GW. Two scenarios for M-GWn: it either is under control of the current C.E. or belongs to another C.E. If M-GWn is under control of the current C.E., C.E. sends a request message to re-route the data path. Otherwise, C.E. does not know the M-GWn. So it has to request MCE to get information, then forward the information to gateways to re-route the data path.

**Figure 9:**
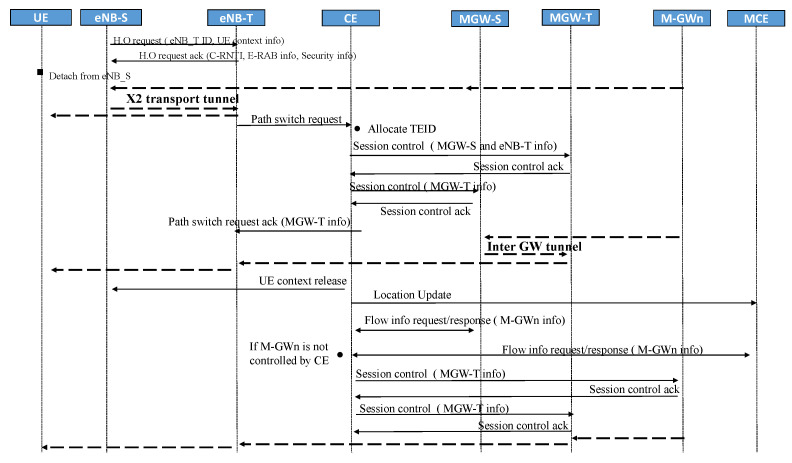
The procedure of the handover with gateway relocation in the proposed 5G concept.

Figure 9 illustrates handover with gateway relocation. After X2 tunnel establishment, the target eNB sends a path switch request to the C.E. entity.Then C.E. determines that the M-GW is relocated (target M-GW); next, C.E. allocates new parameters for the target M-GW. Then it sends a Session control message to the source M-GW and the target M-GW. In the next step, C.E. sends a Path switch to respond to the message from the target M-GW to the target eNB. The target eNB releases the X2 tunnel and creates a GTP tunnel to the target M-GW, and an inter-GW tunnel is also established. C.E. sends the U.E. context release message to inform the handover’s success to the source eNB and triggers the release of resources. In the final step, C.E. requests to the source M-GW or MCE by sending a flow info request message, in which a response with information of M-GWn. C.E. then forwards to M-GWn information about the the target M-GW through the Session control message. Finally, a tunnel between the target M-GW and M-GWn is created to re-route the data packets.

## 4. Numerical Analysis

This section describes the mathematical framework to calculate the transmission delay for the 4G network and the proposed architecture. We consider that a specific message with size S sends between two nodes over the wired link and neglect delay for the wireless link. The following expressions show the signaling cost of the initial attache and handover delay.

We express the transmission delay of the message size *S* transmitted through a wired link from *x* node to *y* node as $C_{x}{,}_{y}\left(S,H_{x\to y}\right)$, where $H_{x\to y}$ donates the number of wired hops. The transmission delay can be denoted as follows:

(1) $$C_{x}{,}_{y}\left(M,H_{x\to y}\right)=H_{x\to y}\left[\frac{S}{B}+D+Q\right]$$

The default parameter and notations used in the performance analysis are described in Table 1.

The initial attachment for the proposed work is described in Section 3.1; this procedure needs two types of messages: the control messages $C_{Initial}^{cn}$ and data massages $C_{Initial}^{d}$. Therefore, the total transmission delay for the initial attache is expressed as follows:

(2) $$\sum C_{Initial}^{cn}+C_{Initial}^{d}$$

the control messages in the proposed architecture can be expressed as follows:

(3) $$C_{Initial}^{cn,5G}=2C_{\delta }+2C_{\epsilon }$$

and the data message

(4) $$C_{Initial}^{d,5G}=2C_{\lambda }$$

On the other hand, the transmission delay for the 4G network for control and data messages can be donated in Equations (5) and (6):

(5) $$C_{Initial}^{cn,4G}=5C_{\delta }+2C_{\gamma }+4C_{\beta }+2C_{\epsilon }$$

(6) $$C_{Initial}^{d,4G}=2C_{\alpha }+2C_{\epsilon }$$

For the handover delay, as described in Section 3.3 and Section 3.4, the handover is divided into the Intra ($C_{Intra}^{HO}$) and Inter ($C_{Inter}^{HO}$). The Intra handover delay can be calculated for 4G network and the proposed network as follows:

(7) $$C_{Intra}^{HO,5G}=2C_{\alpha }+3C_{\delta }+2C_{\beta }+2C\epsilon$$

(8) $$C_{Intra}^{HO,4G}=2C_{\gamma }+2C_{\delta }+5C_{\beta }+C_{\epsilon }+C_{\lambda }$$

and the Inter handover delay expressed as shown in Equations (9) and (10)

(9) $$C_{HO,Inter}^{D,5G}=3C_{\alpha }+2C_{\lambda }+2C_{\epsilon }$$

(10) $$C_{HO,Inter}^{D,4G}=6C_{\delta }+2C_{\beta }+2C_{\epsilon }$$

## 5. Numerical and Simulation Results

### 5.1. Numerical Results

Based on the mathematical expressions given in Section 4, we compare the performance of the 4G network and 5G network architecture by varying the parameters expressed in Table 1.

**Figure 10:**
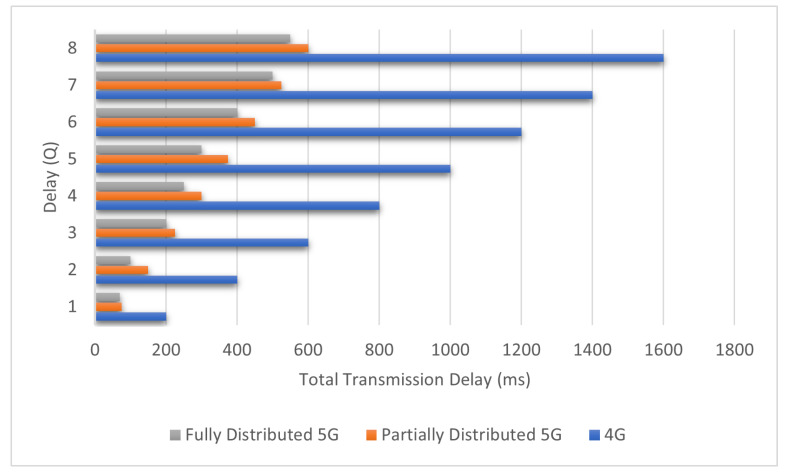
The impact of the queuing delay (Q) at each node.

Figure 10 shows the effect of queuing delay (Q) on cumulative transmission delay at each node in the network. This figure compares three approaches: 4G network, fully distributed, and proposed approach (partially distributed method). The delay increased linearly for all networks. The results show that the proposed method achieves competitive performance. This result is because the data packets in the 5G network are sent through an optimal path—however, the data route in the 4G is delivered by P-GW and S-GW.

**Figure 11:**
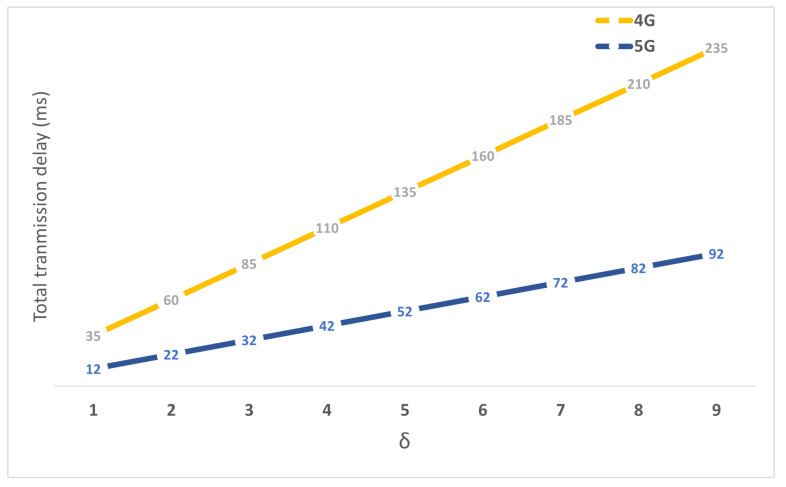
The impact of varying δ on the total transmission delay.

Figure 11 demonstrates the impact of hop count between the eNB and C.E. on the transmission delay by varying δ. The proposed architecture shows superior performance compared with the 4G network due to the optimized route of data packets between the M-GW and eNB instead of a centralized anchor between the S-GW and P-GW in 4G.

**Figure 12:**
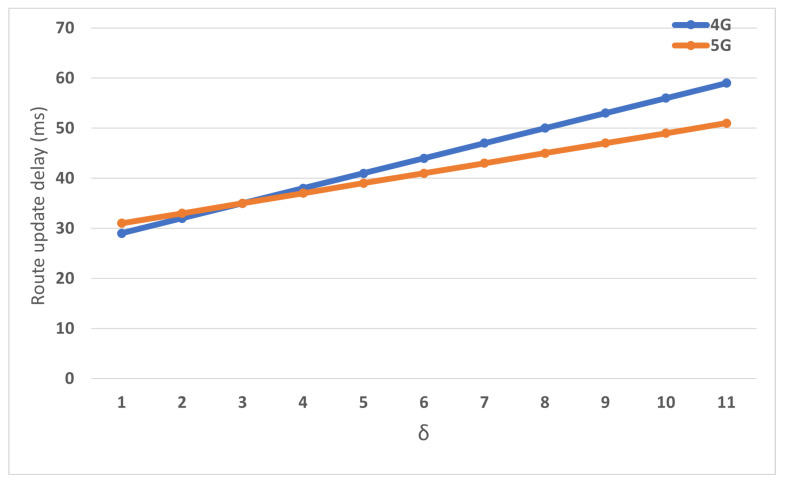
The impact of δ on the handover delay.

Figure 12 compares intra-gateway handover in both networks. The result shows the impact of hop count between eNB and C.E (δ). Varying δ significantly impacted the handover delay for the 4G network. However, the proposed network demonstrates significant enhancement because eNB performs the routing with C.E directly. By contrast, in the 4G network, handover is implemented between the P-GW and MME.

**Figure 13:**
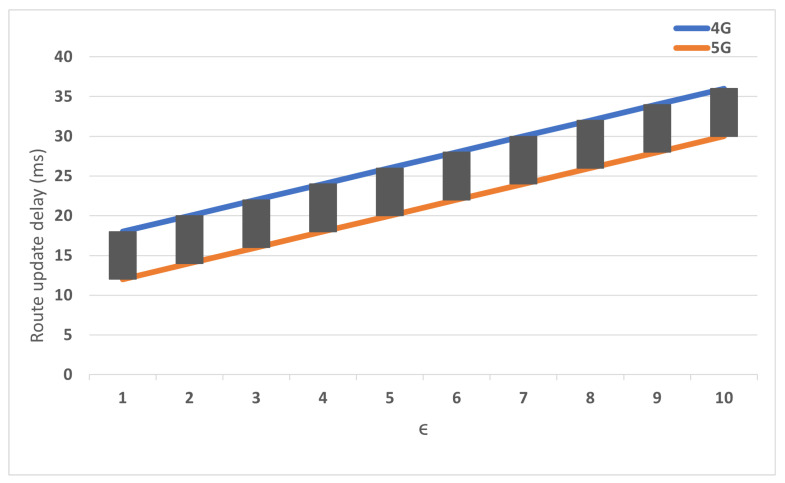
The impact of ϵ on the handover delay.

Figure 13 illustrates the inter-gateway handover delay. The result compares the handover delay between the eNBs in the same network (ϵ). The impact of varying ϵ shows a notable effect on the 4G network delay because the MME changes the bearer via S-GW and P-GW as a centralized anchor. Nevertheless, The 5G architecture is insensitively affected by varying ϵ because the C.E performs path routing with the M-GW.

### 5.2. Simulation Topology

In this section, we evaluate the performance of the proposed model by using the LENA-based NS-3 simulator. LENA NS-3 is open-source code and is available for building a network system. NS-3 has already developed a 4G network topology [48] including two main components: the LTE model and the EPC model. LTE framework is divided into two parts: lower radio protocol stack, which includes particular PHY, MAC layer as well as Scheduler, and the second one is upper radio stack which includes RRC, PDCP, and RLC protocols. These entities within the U.E. and eNB nodes are unmodified because the proposed 5G architecture keeps the access layer of 4G intact. While the EPC framework is modified as follows. First, The S-GW and P-GW are implemented within a single node. Thus, the gateway relocation mobility concept and S5/S8 interface are not supported. Second, the use of a single S-PGW causes incorrect signaling message procedures. Third, the GTP tunneling protocol (GTPv1) was replaced by GTPv2. Finally, socket transmission is used for the data plane (GTP-U) only, and logical connections are used to overcome inaccurate results. We create one more node acted as S-GW to overcome these challenges, then attach mobility functions from the original S-PGW node. Next, we fulfill the GTP protocol by adding GTPv2 features. Then, we make the S5/S8 GTP connection between S-GW and P-GW and implement fully signaling messages. The last step is creating socket transmission for both the control plane (GTP-C) and the data plane (GTP-U), Now the 4G model in NS3 gives more accurate results.

This paper used LENA NS-3 version 3.22 in a Linux environment to build the proposed network topology. The proposed architecture programming consists of, first, building MCE, C.E., M-GW nodes. Second, implementing control and data plane into the corresponding interface. This research focuses on mobility simulations; Intra and Inter-Handover gateway mobility. We compare the results in terms of latency while U.E. moves with different speeds and moves across several domains. The flow monitor module [49] is used to gather and store data from the simulated network. We created a Remote host node that acts as an Internet server that can send packets to other nodes. Additional parameters in the simulation model are shown in Table 2 [50].

### 5.3. Simulation Results

**Figure 14:**
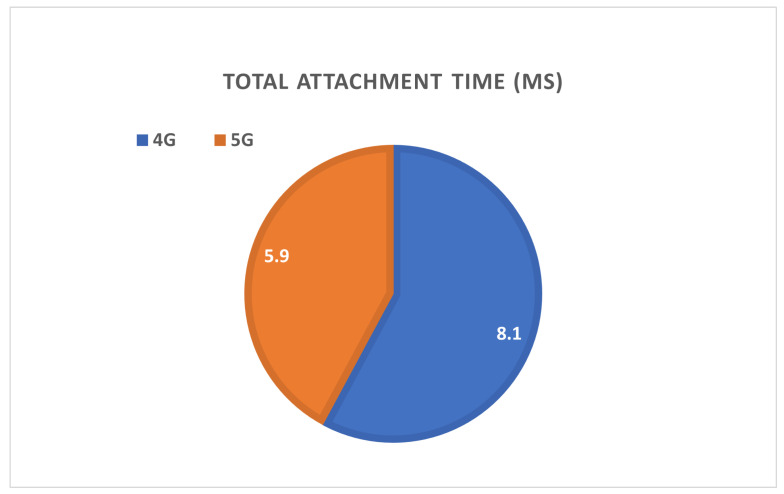
Comparison of 4G and proposed 5G in terms of latency of initial attachment.

At the beginning of our simulation, U.E. has to make an initial attach and establish a GTP tunnel from eNB to gateway. During the handover procedure, the re-attachment steps are similar to the attachment. We simplify the signaling process between eNB and gateway in the new attachment scenario, so the proposed 5G gives a lower average total time. Hence, the total time for re-attachment in case of handover is reduced. Figure 14 compares 4G and proposed 5G in terms of total initial attachment time.

#### 5.3.1. Intra-Gateway Mobility with X2 Handover

**Figure 15:**
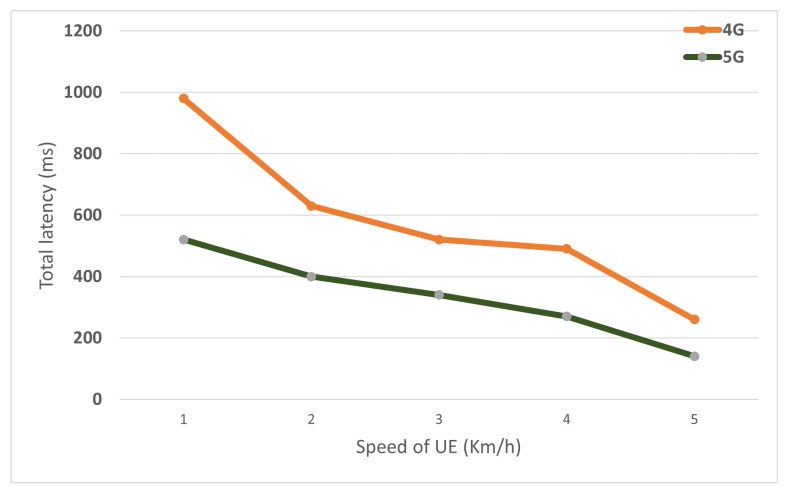
Comparison of 4G and proposed 5G in terms of latency of Intra-Handover with varied UE speed.

As described in the handover section above, Intra-H.O. occurs when U.E. enters a new domain outside the coverage area of the current base station. In the first simulation scenario, U.E. moves from source to target eNB with speed ranging from 18 km/h to 120 km/h. The total execution time is composed of time for attachment/re-attachment and data delivery. The distance between eNBs is 60 m. After the handover procedure finishes successfully, the value delaySum is collected from the flow monitor module. Figure 15 shows the proposed 5G reduces by 40% of total delay time than the legacy 4G architecture for different U.E. speeds. This reflects the ability of the proposed framework to save time and get better results.

**Figure 16:**
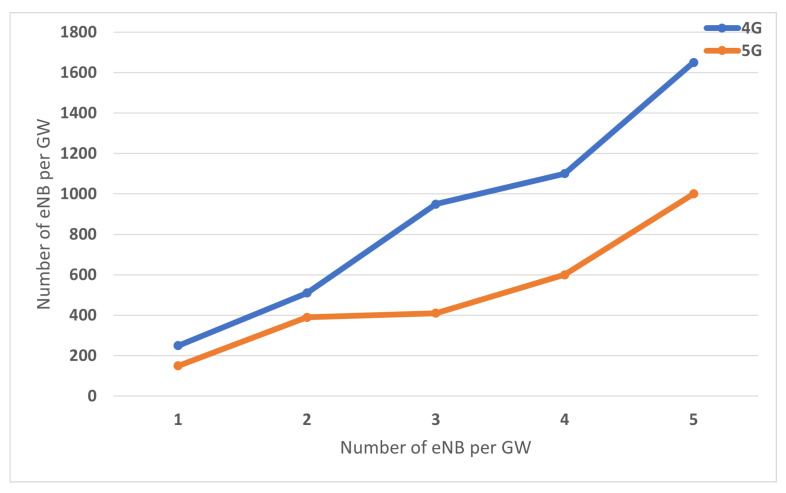
Comparison of 4G and proposed 5G in terms of latency of Intra-Handover with eNB per Gateway.

The second simulation scenario, study the performance while U.E. moves across several eNB in the same gateway’s domain. The number of eNB is varied from 2 to 6. The distance between eNBs is 60 m. All eNBs connect to only one S-GW/ P-GW (in the case of 4G) and one M-GW (proposed 5G). U.E. moves across eNB’s domain with a speed of 108 km/h. After 2 s since U.E. has re-attached to the new eNB, the value delaySum is collected from the flow monitor module. The result is displayed in Figure 16. As expected, the proposed 5G model reduces more than 30% in terms of the total delay time.

#### 5.3.2. Inter-Gateway Mobility with X2 Handover

**Figure 17:**
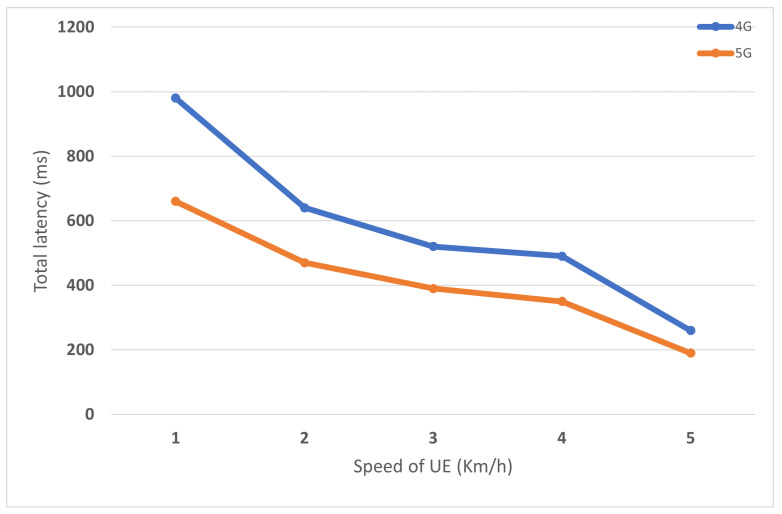
Comparison of 4G and proposed 5G in terms of latency of Inter-Handover with varied UE speed.

**Figure 18:**
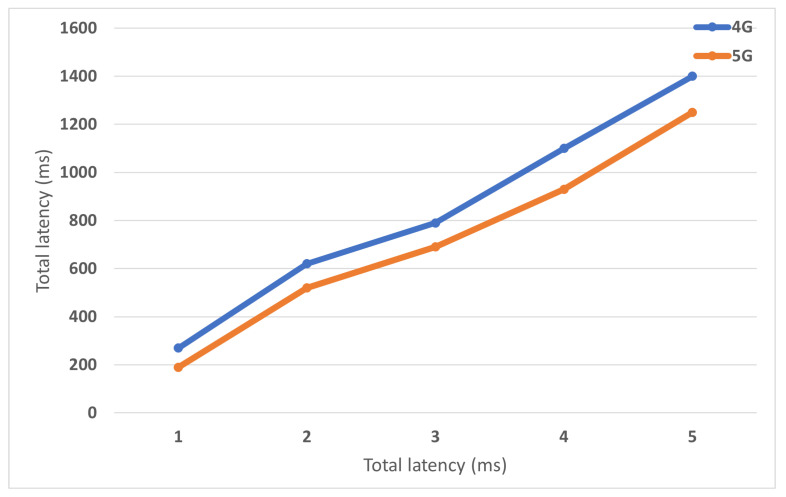
Comparison of 4G and proposed 5G in terms of latency of Inter-Handover with eNB per Gateway.

The Inter-Gateway mobility executes in two-level, X2 tunnel, and Inter-GW tunnel. 5G simulation model is created with several M-GWs; each M-GW attaches to one eNB. For the first simulation scenario, U.E. moves across two gateway’s domain, with different speed values. The result is showed in Figure 17. For the second simulation scenario, U.E. moves across the gateway’s domains, and the Inter-Gateway mobility occurs continuously. As illustrated in Figure 18, the significant impact of optimal routing on performance, compared to 4G that data traffic has to terminate and re-routed at P-GW, proposed 5G with distributed gateway avoids non-optimal path issues improves transmission performance. Compared to the 4G model, the 5G model reduces 27% total delay time in the first simulation scenario and 15% in the second simulation scenario.

## 6. Conclusions

This paper proposes a flat 5G core network with UP distributed architecture to enhance routing, reduce latency, and signaling between various network entities. A novel partially distributed mobility 5G concept is proposed by eliminating centralized anchor gateway and analyzing popular procedures, including initial attach data delivery. We have added several entities to the simulator based on NS-3 and merged other entities’ functionality. We demonstrated that our proposal falls in line with 5G standards and technical reports published by 3GPP. The performance studies show that the mobile packet core can gain a significant benefit to reach the latency demand of 5G. Experiments are conducted on several scenarios using NS-3 simulation to validate the performance of the proposed architecture. Firstly, we build new network entities representing the mobile core, such as Mobile Control Entity, which combines the MME, S-GW, and P-GW, and consists of several control entities to avoid a single point of failure. A new Mobility Gateway acts as anchor mobility inside the network and routing IP packets to the Internet. Secondly, implement control and data plane into the corresponding interface. The proposed SDN-based DMM architecture obtained better results in terms of total transmission delay, inter and intra handover delay, queuing delay, and total attachment time. The Numerical analysis and simulation results showed that the proposed SDN-based DMM architecture performance-enhanced end-to-end latency over the legacy architecture. At the same time, U.E. executes handover procedures faster and moves across several gateways, and third, the proposed 5G architecture delivers data packets with an optimal routing function. We will develop a fully distributed network with complete decoupling of C.P. and UP based on SDN architecture for future work.

## Figures and Tables

**Table 1:** Default values are used in the numerical analysis.

Parameters	Description	Value
D	Delay	2 ms
Q	Queuing delay	2 ms
*cn*	The size of the control messages	50 bytes
*d*	The size of data messages	200 bytes
α	Hop count between eNBs	2
β	Hop count between C.E and M-GW	2
γ	Hop count between HSS and C.E	3
δ	Hop count between eNB and C.E	2
ϵ	Hop count between S-GW and P-GW	3
λ	Hop count between eNB and S-GW	2

**Table 2:** Key parameters for simulation.

Parameter	Setting
Speed of UE	From 5 to 120 km/h
eNB Tx Power	46 dBm
Distance between eNB	100 m
EPS Bearer type	NGBR-VIDEO-TCP
QCI	9
Bandwidth	5 MHz
Data rate	100 Gbps

## Abbreviations

The following abbreviations are used in this manuscript:

4G	Fourth-generation Mobile Network
5G	Fifth-generation Mobile Network
LTE	Long-Term Evolution
EPS	Evolved Packet System
E-UTRAN	Evolved Universal Terrestrial Radio Access Network
EPC	Evolved Packet Core
eNB	eNodeB
MME	Management Mobility Entity
S-GW	Serving Gateway
P-GW	Packet-data-network gateway
HSS	Home Subscription Server
UE	User Equipment
GPRS	General Packet Radio Service
GTP	GPRS tunneling protocol
SDN	Software Defined Networking
DMM	Distributed Mobility Management
PMIPv6	Proxy Mobile IPv6
MMO	Multi-Objective optimization
R.A	Ratio Analysis
UP	User plane
C.P.	Control plane
C.E.	Control Entity
MCE	Mobile Control Entity
TEID	Tunnel Endpoint Identifier
QCI	Quality Class Identifier
GBR	Guaranteed Bit Rate
TAU	Tracking Area Updates
M-GW	Mobility Gateway
PDN	Packet Data Network
IMSI	International Mobile Subscriber Identity
TAI	Tracking Area Identifier
ECGI	E-UTRAN Cell Global Identifier
TEID	Tunnel Endpoint IDentifier
H.O.	Handover
Inter-GW H.O.	Inter Gateway Handover
Intra-Gw H.O.	Intra Gateway Handover
